# Logic regression-derived algorithms for syndromic management of vaginal infections

**DOI:** 10.1186/s12911-015-0228-5

**Published:** 2015-12-16

**Authors:** Sujit D. Rathod, Tan Li, Jeffrey D. Klausner, Alan Hubbard, Arthur L. Reingold, Purnima Madhivanan

**Affiliations:** Department of Population Health, London School of Hygiene and Tropical Medicine, London, UK; Department of Biostatistics, Florida International University, Miami, FL USA; Division of Infectious Diseases, University of California, Los Angeles, CA USA; Division of Epidemiology, University of California, Berkeley, CA USA; Public Health Research Institute of India, Mysore, India

**Keywords:** Sensitivity and specificity, India, Vaginitis, Epidemiologic methods, Humans, Female, Regression analysis, Algorithms

## Abstract

**Background:**

Syndromic management of vaginal infections is known to have poor diagnostic accuracy. Logic regression is a machine-learning procedure which allows for the identification of combinations of variables to predict an outcome, such as the presence of a vaginal infection.

**Methods:**

We used logic regression to develop predictive models for syndromic management of vaginal infection among symptomatic, reproductive-age women in south India. We assessed the positive predictive values, negative predictive values, sensitivities and specificities of the logic regression procedure and a standard WHO algorithm against laboratory-confirmed diagnoses of two conditions: metronidazole-sensitive vaginitis [bacterial vaginosis or trichomoniasis (BV/TV)], and vulvovaginal candidiasis (VVC).

**Results:**

The logic regression procedure created algorithms which had a mean positive predictive value of 61 % and negative predictive value of 80 % for management of BV/TV, and a mean positive predictive value of 26 % and negative predictive value of 98 % for management of VVC. The results using the WHO algorithm were similarly mixed.

**Conclusions:**

The logic regression procedure identified the most predictive measures for management of vaginal infections from the candidate clinical and laboratory measures. However, the procedure provided further evidence as to the limits of syndromic management for vaginal infections using currently available clinical measures.

**Electronic supplementary material:**

The online version of this article (doi:10.1186/s12911-015-0228-5) contains supplementary material, which is available to authorized users.

## Background

In south India, previous research has established high levels of reproductive tract symptoms – particularly of vaginal discharge - in spite of the low prevalence of *Chlamydia trachomatis* and *Neisseria gonorroheae* infections [1–9]. Bacterial vaginosis, *Trichomonas vaginalis* and *Candida* species are frequently implicated as the cause of the symptoms. These vaginal infections are thought to increase risk of infection by HIV and adverse birth outcomes, and to be responsible for substantial psychological distress and economic costs [10–12].

Due to the unavailability of inexpensive point-of-care diagnostic tests, syndromic management continues to remain the primary means addressing such conditions in low-income settings. Syndromic algorithms are commonly used to diagnose reproductive tract infections in women presenting with symptoms in these settings, so that they can be seen and treated in a single visit.

Validation studies in low-resource settings have shown the specificity of established syndromic algorithms – such as those developed by the World Health Organization (WHO) [13] - for vaginal infections (i.e. bacterial vaginosis, trichomoniasis and vulvovaginal candidiasis) to be around or below 50 % [14–19]. The correspondingly low positive predictive values of syndromic algorithms indicate that the use of the algorithms leads to substantial over-diagnosis, overtreatment with antibiotics, and an increase in the average cost per true case treated [17]. Attempts have been made to improve on the established syndromic algorithms, with modest improvement, at best, in diagnostic accuracy [14, 17].

Logic regression – not to be confused with *logistic* regression - is a nonparametric statistical method which has been used to identify combinations of binary measures via Boolean (logic) combinations (e.g. (A AND B) OR NOT C)) to optimally predict an outcome [20]. When measures (such as those collected from a clinical interview) are predictive of a disease condition, logic regression uses the measures in various combinations to create a syndromic algorithm which is evaluated as being TRUE (the condition is presumed to be present) or FALSE (the condition is presumed to be absent). In contrast to other machine-learning processes for prediction, logic regression produces potentially simple decision rules, which are appropriate for implementation in a low-resource clinical setting. Logic regression was initially developed and applied to explore high-order interactions of single nucleotide polymorphisms with disease outcomes. More recently, logic regression has been employed to use biospecimen data to predict a range of clinical outcomes [21] and to use sociodemographic and behavioural characteristics to pre-screen individuals at high-risk for colorectal cancer [22]. Further details concerning logic regression’s fitting procedures and performance in comparison to other machine-learning processes are available [21, 23].

Here, the logic regression method was used to develop predictive models for syndromic management of two vaginal conditions: metronidazole-sensitive vaginitis (bacterial vaginosis or trichomoniasis), and vulvovaginal candidiasis. The logic regression models and the standard WHO algorithm were assessed for their accuracy (i.e. positive predictive value, negative predictive value, sensitivity and specificity) against laboratory-confirmed vaginal infections among reproductive-age women in south India.

## Methods

The Prerana dataset was collected as part of a six-month prospective cohort study to examine the relationship of lower genital tract infections and incident Herpes simplex virus type 2 infection among women living around Mysore, India in 2005–6. The methods used to recruit the 898 women in the cohort have been described elsewhere [24]. Briefly, women were recruited from outpatient clinics and women’s self-help groups in the peri-urban and rural areas around Mysore. Eligibility criteria were: age between 15 and 30 years, sexually active (defined as having had vaginal intercourse at least once in the three months prior to recruitment), planning on residing in the area for at least six months, and willing to undergo study procedures. Women who were pregnant or had vaginal bleeding were excluded from the study. Eligible women who expressed interest in participation provided written informed consent for the study at the time of enrolment. The same informed consent process applied to married women under 18 years of age, who were eligible to participate as emancipated minors. Study visits were completed at baseline, and follow-up visits three and six months later. The study visits involved an interviewer-administered questionnaire in *Urdu* or *Kannada*; a pelvic examination conducted by a female study physician; and collection of vaginal specimens and blood for laboratory testing. This study procedure, including the informed consent process for emancipated minors, was approved by our IRBs: the Committee for Protection of Human Subjects at the University of California, Berkeley and the Asha Kiran Hospital Institutional Review Board.

### Interview and clinical evaluation

The interviewer-administered questionnaire collected information concerning sociodemographic characteristics; sexual and reproductive health; history and current complaints of abnormal vaginal symptoms; and sexual partner characteristics. During pelvic examination, the study physician recorded the absence or presence of abnormal vaginal findings and collected vaginal fluid samples from the posterior fornix of the vagina.

### Laboratory tests and diagnoses

Laboratory testing was conducted at the Vikram Hospital laboratory in Mysore. Vaginal swab samples were used for pH testing (SD fine chemicals Ltd, Mumbai, India), Gram stain of vaginal smear, and saline wet mount microscopy to detect clue cells or motile trichomonads. The presence of amines was evaluated by sniffing a drop of KOH on a vaginal swab (whiff test). The vaginal swab samples were also used to culture *T. vaginalis* (InPouch, BioMed Diagnostics, White City, OR, USA) and *Candida* (InTray Colorex Yeast, BioMed Diagnostics). There was independent verification of 10 % of tests for *T. vaginalis* and *Candida* by a second microbiologist.

A laboratory diagnosis of bacterial vaginosis was made using the Gram stain scoring criteria developed by Nugent et al. a total score of 7–10 was considered consistent with bacterial vaginosis [25], which was assessed by two independent laboratory technicians. A diagnosis of trichomoniasis was made if motile trichomonads were detected on microscopy from a vaginal swab specimen or on culture within five days. Women were considered to be colonized by *Candida* if positive on culture. A diagnosis of vulvovaginal candidiasis was made if, in addition to *Candida* colonization, women reported at least one of two vaginal symptoms (itching or discharge) in the interview and the study clinician observed at least one of two vaginal signs (erythema or discharge) during the pelvic examination.

Women were treated per United States Centers for Disease Control and Prevention guidelines [26] if clinically diagnosed with bacterial vaginosis, or if they had a diagnosis of *T. vaginalis* infection or vulvovaginal candidiasis. Treatment was offered to the women and their sex partners upon diagnosis of trichomoniasis.

### Statistical methods

Two conditions were considered as outcomes for two separate evaluations of syndromic algorithms: 1) laboratory-confirmed bacterial vaginosis or trichomoniasis (BV/TV); and 2) vulvovaginal candidiasis (VVC). As the presence of bacterial vaginosis and *T. vaginalis* infection have similar clinical presentation, are commonly present together, and can be treated with the same antibiotic regimen, they were considered as a single condition here: metronidazole-sensitive vaginitis. In addition, the WHO algorithm we evaluated treats both conditions similarly in practice [13].

As participants could contribute up to three observations to the cohort dataset and the logic regression procedure assumes independent observations, we selected one observation per participant for each of the two analysis datasets (one for BV/TV, one for VVC). To create each dataset, we use the following steps: We eliminated all observations for which a participant did not report one or more symptom associated with vaginal infection (i.e. vaginal itching, discharge or burning). These symptoms predominate among women who opt to visit a health clinic for treatment and are used as the entry point for users of the WHO algorithm. Thus the same set of observations will be included for consideration by the logic regression procedure. To select one observation per participant for the BV/TV analysis, we drew from all three study visits. Among women who tested positive for laboratory-confirmed BV/TV at any of the three study visit, only the first visit at which the participant was positive was included in the BV/TV dataset. Among women who were negative at all visits, only the baseline visit was included in the BV/TV dataset. To select one observation per participant for the VVC analysis, we drew from the latter two study visits. At those latter visits, study physicians consistently recorded the presence or absence of curd-like vaginal discharge, which is thought to be highly suggestive of VVC. Among women who were tested positive for laboratory-confirmed VVC at any of the latter two study visits, the first visit at which the participant was positive was included in the VVC dataset. Among women who were negative at both latter visits, only the three-month study visit was included in the VVC dataset.

**Fig. 1:**
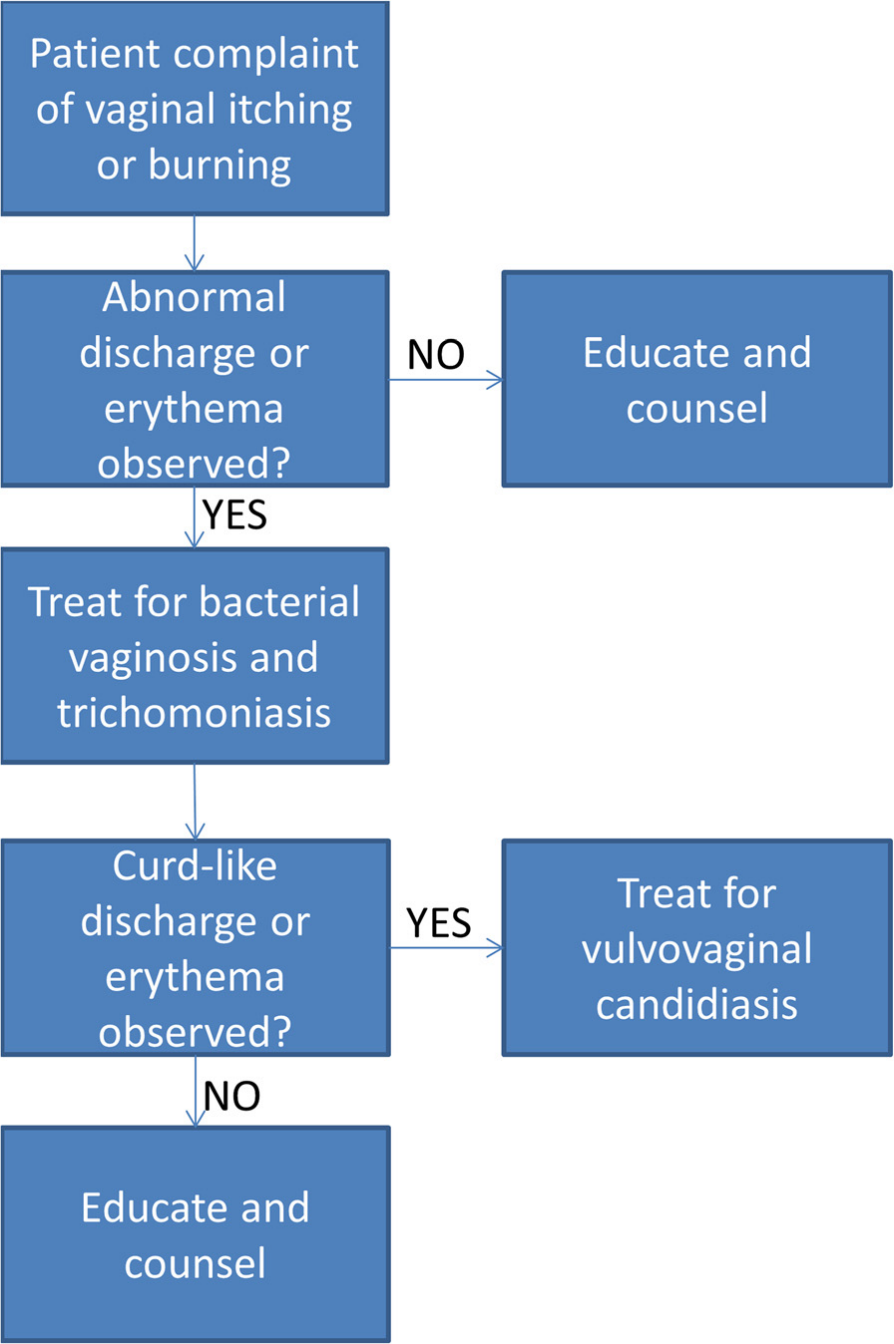
Modified WHO algorithm for syndromic diagnosis of bacterial vaginosis, trichomoniasis, or vulvovaginal candidiasis

Using these two datasets, the standard WHO algorithm for syndromic management of reproductive tract infections was evaluated against laboratory-confirmed diagnoses of BV/TV and of VVC. The WHO algorithm used here allows for use of measures collected from a clinical interview and a non-invasive pelvic examination; it was modified to make diagnoses of vaginal infections only, rather than both vaginal and cervical infections (i.e. *N. gonorrhoeae* and *C. trachomatis*) (Fig. 1). For each outcome the WHO algorithm was applied to the same subsets of observations in which women reported one or more symptom associated with vaginal infection (i.e. vaginal itching, discharge or burning). The syndromic algorithms were compared to the laboratory-confirmed diagnoses of BV/TV and of VVC to calculate diagnostic accuracy figures (i.e. positive and negative predictive values, and sensitivity and specificity), their respective standard errors and 95 % confidence intervals.

Next, the logic regression procedure was used to create syndromic diagnostic algorithms for BV/TV and for VVC, and each procedure was evaluated for accuracy. Candidate variables for logic models were all binary measurements, and included women’s reports of vaginal symptoms (i.e. report of current itching, burning, discharge, and discharge present in the past three months); vaginal features observable as part of the pelvic examination (i.e. presence or absence of vaginal erythema or abnormal discharge); the findings from laboratory testing of vaginal specimens (i.e. pH ≥ 4.5, positive whiff test); and a sex partner characteristic (i.e. whether he may have additional sex partners) (Table 1). The presence or absence of curd-like discharge was only considered for the logic regression model for diagnosis of VVC. The selection of the aforementioned candidate variables was guided by their inclusion on the WHO algorithm, and was supplemented by two other measures (i.e. pH and whiff tests) that have been demonstrated to involve non-invasive vaginal specimen collection and simple, low-cost testing procedures [27].

**Table 1:** Measures selected for evaluation of the WHO algorithm for syndromic management of vaginal infections among symptomatic reproductive-age women, and for inclusion for the logic regression procedure, Mysore, India, 2005–2006

Measures on the WHO algorithm	
	Vaginal discharge reported by participant
	Vulval itching reported by participant
	Burning reported by participant
	Abnormal vaginal discharge observed by clinician
	Curd-like vaginal discharge observed by clinician^a^
	Vulval erythema observed by clinician
Additional symptoms and behaviors included for logic regression	
	Discharge in the past three months reported by participant
	Sex partner may have other partners reported by participant
Additional signs and laboratory tests included for logic regression	
	Vaginal pH ≥ 4.5
	Whiff (KOH) test for presence of vaginal amines

^a^Only assessed at the three- and six-month study visits

The estimation of the diagnostic accuracy from logic regression derived models was done with external V-fold cross-validation, to decrease bias in estimation. The cross-validation procedure divides the analysis dataset randomly into equal-sized subsets, stratified so cases are distributed proportionally across subsets. For each cross-validation run, V-1 subsets are used as a training dataset to select a logic model, which is then applied to the testing dataset to calculate its diagnostic accuracy.

For each training dataset, a single logic model was selected out of several candidate models through consideration of results from internal cross-validation and permutation testing. These results are ranked according to the predictive error of each model, such that models with lower total error in positive and negative predictive values were stronger candidates for selection. When multiple candidate logic models appeared to have optimal results within a single training run, the model using the fewest variables was selected, given the preference for parsimonious diagnostic algorithms for potential use by clinicians in low-resource settings. The logic model selected from the training dataset was then applied to the testing dataset to estimate the diagnostic accuracy. The mean of the V diagnostic accuracy figures were calculated and reported here. The standard errors for the figures - which are equivalent to the standard deviations - are also reported with the 95 % confidence intervals. The analysis was completed using R 2.13.2 (R Foundation for Statistical Computing, Vienna, Austria), the LogicReg package 1.4.14 [28] and Stata 11.2 (StataCorp, College Station, USA). The R code used for this analysis is available as Additional file 1.

## Results

The 898 women in the cohort have been described in detail, as has the baseline prevalence of signs and symptoms associated with reproductive tract infections [27, 29]. Briefly, the 898 participants had a median age of 26 (IQR 24–29); 69 % were Hindu, 29 % were Muslim and 2 % were Christian; 27 % had no formal education; 98 % were married (including 100 % of women under 18 years of age) and another 1 % was living with a partner; 85 % had at least one child; and 0 % tested positive for *N. gonorrhoeae* at the baseline visit. The 898 participants completed 2551 study visits over 6 months, of which 777 visits involved report of at least one symptom associated with vaginal infection. For the BV/TV dataset, we selected 443 unique participants from the 777 observations – of whom 117/443 (26 %) were positive for BV/TV. The observations in this dataset were allocated into 10 folds for cross-validation, and for evaluation using the WHO algorithm. For the VVC dataset we selected 227 unique participants from the 777 observations – of whom 45/227 (20 %) were positive for VVC. The observations were allocated into five folds for cross-validation, and for evaluation using the WHO algorithm.

Using the logic regression cross-validation procedure on the ten BV/TV training datasets, for nine of the ten logic models a positive whiff test was sufficient for syndromic diagnosis of BV/TV. In one cross-validation run, a model consisting of six measurements (i.e. ((positive whiff AND high pH) OR (vaginal erythema observed by clinical exam AND vulval itching reported by participant)) OR (vaginal discharge reported by participant AND positive whiff test)) was selected. For five VVC training datasets, all five models selected from the cross-validation procedure were comprised only of the clinical assessment of vaginal discharge, whereby the clinician’s observation of abnormal vaginal discharge was sufficient for a syndromic diagnosis of VVC.

The positive predictive values, negative predictive values, sensitivities, specificities and the corresponding 95 % confidence intervals for diagnosis of BV/TV and of VVC using the WHO algorithm and the logic regression procedure are reported in Table 2.

**Table 2:** Accuracy of the WHO algorithm and the logic regression procedure for management of vaginal infections: Mysore, India 2005–2006

Vaginal infection(s) and algorithm	PPV (SE)	95 % CI	NPV (SE)	95 % CI	Se (SE)	95 % CI	Sp (SE)	95 % CI
Bacterial vaginosis or Trichomoniasis (Prevalence = 117/443, 26 %)								
WHO algorithm	32 (3)	25, 39	78 (3)	73, 83	53 (5)	44, 62	60 (3)	54, 65
Logic model	61 (18)	25, 98	80 (4)	72, 87	33 (14)	6, 60	93 (4)	85, 100
Vulvovaginal candidiasis (Prevalence = 45/227, 20 %)								
WHO algorithm	44 (6)	32, 57	90 (2)	84, 94	64 (7)	49, 78	80 (3)	73, 85
Logic model	26 (2)	22, 31	98 (2)	93, 100	93 (6)	82, 100	64 (4)	56, 73

*PPV* positive predictive value, *NPV* negative predictive value, *Se* sensitivity, *Sp* specificity, *SE* standard error, *CI* confidence interval

## Discussion

This is the first evaluation we are aware of the logic regression procedure for management of vaginal infections. We used logic regression to identify a combination of symptoms and features recorded from a pelvic examination to predict the presence of bacterial vaginosis or trichomoniasis, or vulvovaginal candidiasis. The logic regression procedure was run using a cross-validation procedure which was designed to maximize the positive and negative predictive values for diagnostic models. The selected models produced by logic regression were easily interpretable as diagnostic algorithms, much in the manner that the WHO algorithm is currently used. Both the logic regression models and the WHO algorithm had mixed results: Our results from logic regression indicate that a single measurement (whiff test) generally offers the best prediction for diagnosis of BV/TV, with only marginal improvement through use of more complex logic models. For diagnosis of BV/TV the logic regression procedure was superior to the WHO algorithm on both the positive predictive value (61 to 32 %, respectively) and the negative predictive value (80 to 78 %, respectively). For management of VVC, again, a logic model consisting of a single measurement (abnormal vaginal discharge observed by clinician) offered similar performance as more complex logic models. In this case, the logic regression procedure was inferior to the WHO algorithm on the positive predictive value (26 to 44 %, respectively) though superior on the negative predictive value (98 to 90 %).

One notable outcome of this analysis was identification of the whiff test as being highly specific for management of BV/TV, and the clinician’s observation of vaginal discharge was highly sensitive for management of VVC. Neither result was unsurprising, as the whiff test is an element of the clinical diagnosis for BV [30], and clinical observation of vaginal discharge is part of our case definition for VVC. Though we used several additional clinical measures beyond those included in the WHO algorithm, the logic regression procedure did not produce evidence that any combination of measurements were strongly predictive of the presence of vaginal infections. Prior research has provided a growing body of evidence as to the limited utility of signs and symptoms for syndromic management of reproductive tract infections [14–19, 27]; our results are consistent in this regard.

We used a default scoring function for logic regression, whereby the logic model is nested into a logistic regression equation, and different models are assessed according to the model deviance (i.e. difference of predicted outcome vs the gold-standard outcome). This scoring function serves to maximize the positive and negative predictive values of a given logic model. Future work using logic regression can consider developing a user-defined scoring function to maximize sensitivity and specificity instead. Another potential scoring function could attempt to minimize the cost per true case detected, which would be of particular relevance in low-resource settings. We opted to limit our analysis to a single logic regression tree; the LogReg program is capable of developing algorithms using several trees, use of which should improve results, though with a trade-off of additional complexity in making a diagnosis. Those seeking to maximize predictive performance should consider using multiple trees and a different scoring function such as the Briar Score.

Though we used logic regression’s cross-validation procedure to select our final logic models, these models require additional validation in a similar population of women reporting symptoms associated with vaginal infections. Our results may not be generalizable to populations of women with differing prevalences of other reproductive tract infections – particularly those which cause abnormal discharge - or to women who are more or less likely to be aware of and report vaginal symptoms. Syndromic management requires accurate reporting of symptoms to clinicians; in particular, the validity of reports of vaginal discharge in south Asia has been called into question [19, 31]. Therefore, if a logic regression-derived model is to be used to develop a syndromic management algorithm in a new population it must be validated against a gold-standard diagnosis and must consider cultural aspects of reporting symptoms.

## Conclusion

We used logic regression to create algorithms for syndromic management of vaginal infections; the logic regression procedure ultimately identified known predictors for two vaginal conditions. In this case, the logic regression procedure provided evidence as to the limits of using currently available clinical measures for syndromic management for vaginal infections. The methods described here can be extended to other health conditions to identify a combination of predictors collected from a clinical history, examination or laboratory testing. That the logic regression program is available at no cost and can be implemented with user-generated scoring functions makes it an appealing option for use in low-income settings.

## Additional file

Additional file 1: R code for logic regression-derived algorithms for syndromic diagnosis of vaginal infections. (TXT 4 kb)
